# Results of a phase I dose escalation study of eltrombopag in patients with advanced soft tissue sarcoma receiving doxorubicin and ifosfamide

**DOI:** 10.1186/1471-2407-13-121

**Published:** 2013-03-16

**Authors:** Sant P Chawla, Arthur Staddon, Andrew Hendifar, Conrad A Messam, Rita Patwardhan, Yasser Mostafa Kamel

**Affiliations:** 1 Sarcoma Oncology Center, Santa Monica, CA, USA; 2 University of Pennsylvania School of Medicine, Pennsylvania Hospital, Philadelphia, PA, USA; 3 GlaxoSmithKline, 1250 S. Collegeville Road, Collegeville, PA, USA; 4 GlaxoSmithKline, Stockley Park, UK

**Keywords:** Sarcoma, Platelets, Thrombopoietin receptor agonists, Chemotherapy, Myelosuppression, Chemotherapy-induced thrombocytopenia

## Abstract

**Background:**

The objective of this Phase I dose escalation study was to explore the safety and tolerability of eltrombopag, an oral, nonpeptide, thrombopoietin receptor agonist, in patients with advanced soft tissue sarcoma (STS) and thrombocytopenia due to treatment with doxorubicin and ifosfamide (AI) combination chemotherapy.

**Methods:**

Patients aged 18 or older with histologically confirmed, locally advanced or metastatic STS were treated with 1 cycle of AI followed by AI with eltrombopag starting at Cycle 2, using 2 different dosing schedules. The study design included an eltrombopag dose escalation phase starting at 75 mg daily to determine the optimal biological dose (OBD).

**Results:**

Eighteen patients were enrolled and 15 received at least 1 dose of chemotherapy; 3 patients withdrew prior to receiving eltrombopag. Seven, 4, and 1 patients received 75 mg, 100 mg, and 150 mg eltrombopag daily, respectively. No dose-limiting toxicities were reported. Due to slow recruitment, the study was closed prior to identifying an OBD. The most common hematologic adverse events (AEs) were thrombocytopenia (80%), neutropenia (73%), and anemia (67%). The most common nonhematologic AEs were fatigue (53%), alanine aminotransferase increased, constipation, and nausea (47% each). Eleven of 12 patients who received eltrombopag completed at least 2 chemotherapy cycles; all had increased platelet counts on Day 1 of Cycle 2 (cycle with eltrombopag) compared to Day 1 of Cycle 1 (cycle without eltrombopag).

**Conclusions:**

Although data are limited, safety data were consistent with the known toxicities of AI combination chemotherapy or the side effect profile of eltrombopag seen in other studies. Available data suggest a potential pre- and post-chemotherapy dosing scheme for eltrombopag when administered with AI chemotherapy, and support further investigation of eltrombopag treatment in patients with chemotherapy-induced thrombocytopenia.

## Background

Thrombocytopenia is a common treatment-related Grade 3/4 adverse event (AE) and dose-limiting toxicity for various chemotherapy regimens [1-4]. Doxorubicin and ifosfamide, alone and in combination (AI), are active in the treatment of soft tissue sarcomas (STS), with demonstrated positive response rates and improvements in overall survival; however, both agents have been associated with Grade 3/4 thrombocytopenia that is cumulative with successive chemotherapy cycles [5-10].

Chemotherapy-induced thrombocytopenia (CIT) may lead to dose reductions or dose delays, resulting in less than optimal disease control. In severe cases, CIT may result in hemorrhage and a need for platelet transfusions, which have cost and safety limitations [6,8,9,11]. Although interleukin-11 (IL-11), a hematopoietic growth factor with thrombopoietic activity, is approved for the treatment of CIT in the US, it is not approved in the EU, it has modest efficacy, and it produces substantial adverse effects that limit its use [12-14].

Eltrombopag, an oral, nonpeptide, thrombopoietin receptor agonist, increases platelet counts in adult patients with chronic immune thrombocytopenia (ITP) [15-19] and chronic liver disease due to hepatitis C virus infection [20,21].

A Phase II, multicenter, placebo-controlled study tested 3 different doses of eltrombopag vs placebo in patients with solid tumors receiving carboplatin and paclitaxel chemotherapy. The study demonstrated that eltrombopag administration for 10 days post-chemotherapy administration on Day 1 resulted in increased platelet counts starting at Day 8 compared to placebo, with peak platelet counts reached between Day 18 and Day 22 [22]. The study did not meet its primary endpoint of reducing the platelet count change from Day 1 of Cycle 2 to the platelet nadir of Cycle 2, compared to placebo [22].

Thrombocytopenia remains an important clinical problem in the treatment of cancer. As such, this study explored the safety and tolerability of eltrombopag administered according to 2 dosing schedules in patients with advanced STS treated with the AI chemotherapy regimen.

## Methods

### Study design

The primary objective of this Phase I study was to determine the safety and tolerability of eltrombopag in patients with locally advanced or metastatic STS receiving combination chemotherapy with AI. Secondary objectives were to determine the optimal biological dose (OBD), pharmacokinetics (PK), and pharmacodynamics (PD) of eltrombopag in these patients; and to evaluate the impact of eltrombopag on the PK of doxorubicin and doxorubicinol in this treatment setting.

The study protocol, any amendments, informed consent, and other information that required pre-approval were reviewed and approved by the sites where patients were recruited into the study: Western Institutional Review Board, Olympia, WA, USA; Institutional Review Board. Pennsylvania Hospital, Philadelphia, PA, USA; and the University of Texas, M. D. Anderson Cancer Center, Surveillance Committee FWA-363, Houston, TX, USA. This study was conducted in accordance with the International Conference on Harmonisation’s Guidelines for Good Clinical Practice (ICH GCP) and all applicable patient privacy requirements, and the ethical principles that are outlined in the Declaration of Helsinki. This study is registered at http://www.clinicaltrials.gov (NCT00358540).

All participants provided informed consent prior to performance of any study-specific procedures. All patients were scheduled to receive 10 days of eltrombopag dosing starting in Cycle 2, either continuously for 10 days following AI chemotherapy (Days 5 to 14) or for 5 days before (Days -5 to -1) and 5 days after (Days 5 to 9) AI chemotherapy (Days 1 to 4). Each cycle consisted of 21 days. Doxorubicin was administered as a 75 mg/m^2^ intravenous (IV) bolus (Day 1) or as 3 consecutive 25 mg/m^2^ IV boluses (Days 1 to 3); ifosfamide was administered as a 2.5 g/m^2^ IV infusion for 4 days (Days 1 to 4). Mesna and dexrazoxane were administered as per the current standard of care.

The original study design included 2 components: a dose-escalation phase to determine the OBD of eltrombopag in combination with AI, with a daily starting dose of 75 mg eltrombopag and escalating in a stepwise fashion to 100 mg, 150 mg, 200 mg, 250 mg, and 300 mg daily; and an expansion phase to enroll additional patients at the OBD to a maximum of 48 total patients, in order to further explore the efficacy of eltrombopag in this patient population. Due to slow recruitment, dose escalation was halted at the 150-mg dose level prior to identification of an OBD, the expansion phase was not initiated, and the study was closed prior to completion.

Study completion was defined as receiving ≥ 1 dose of eltrombopag starting from Cycle 2 and completing all visits through to the end of Cycle 2. Patients were permitted to stay on study for up to 6 cycles of chemotherapy (5 cycles of eltrombopag dosing).

### Patient selection

Eligible patients were age 18 or older with histologically confirmed, locally advanced or metastatic STS; an Eastern Cooperative Oncology Group (ECOG)-Zubrod performance status of 0 or 1; adequate hematologic, hepatic, and renal function; a life expectancy of ≥ 3 months; no history of platelet disorders or dysfunction, or bleeding disorders; and were otherwise candidates for AI chemotherapy.

Study enrollment was initially limited to chemotherapy-naïve patients. A protocol amendment (January 2008) during the active enrollment period allowed enrollment of patients with 0 or 1 previous chemotherapy regimens and required that all patients had developed ≥ Grade 2 thrombocytopenia (platelet nadir ≤ 75,000/μL) in a previous chemotherapy treatment setting. Alternatively, patients with no previous chemotherapy treatment should have developed ≥ Grade 2 thrombocytopenia during a previous AI chemotherapy cycle, with AI at the same dose and schedule planned in the 2 cycles following enrollment into the study. An additional change in this amendment allowed enrollment for patients with thromboembolic events (TEEs) > 6 months previously; prior to this amendment patients with a history of TEEs were excluded from the study. A subsequent (July 2009) protocol amendment required that patients have adequate cardiac function at baseline, as measured by echocardiogram (ECHO) or multiple gated acquisition (MUGA) scan, as newly available in vitro data demonstrated that eltrombopag was an inhibitor of breast cancer resistance protein (BCRP) efflux transporter, for which doxorubicin and potentially its metabolite, doxorubicinol, are substrates. As these findings suggested that eltrombopag had the potential to increase doxorubicin(ol) plasma concentrations, the protocol was amended to implement additional cardiac monitoring and PK sampling for doxorubicin(ol).

Patients were excluded if they had > 1 previous chemotherapy regimens in any disease setting; preexisting cardiovascular disease; any known clotting disorder associated with hypercoagulability; prior treatment that affected platelet function or anticoagulants for > 3 consecutive days within 2 weeks of the study start and until the end of the study; recent history of drug-induced thrombocytopenia; history of prior radiotherapy (RT) to more than 20% bone marrow bearing sites; planned cataract surgery; or any clinical abnormality or laboratory parameters that interfered with study treatment or conferred a risk for participation in the study.

### Study assessments, procedures, and analyses

Assessments performed at screening (within 14 days prior to the first cycle of treatment) included evaluation of eligibility criteria; medical history; routine physical examination; ECOG performance status; risk factors for kidney impairment and cataracts; 12-lead electrocardiogram (ECG); laboratory assessments (hematology with complete blood count, serum chemistries, urinalysis, and renal assessments); and ophthalmologic examination. The July 2009 amendment required cardiac monitoring using ECHO or MUGA scans at baseline and every 3 cycles.

Physical examinations were performed on study Day 1 of each cycle and on the last day of Cycle 6 or upon withdrawal from study. Ophthalmic assessment was performed at study completion/withdrawal and also at the 6-month follow-up visit. Bleeding events, AE/toxicity assessment, and concomitant medications were assessed at each study visit and on the last day of Cycle 6 or upon withdrawal from the study. Additional safety assessments (renal assessments, ECG recordings, hematology assessments, and chemistry assessments) were completed throughout the study at protocol-specified time points.

Safety and efficacy analyses were summarized by descriptive statistics. Safety analyses were reported using the safety population, comprising all patients who received ≥ 1 dose of AI chemotherapy. Efficacy analyses were reported using the efficacy population, comprising all patients who received ≥ 1 dose of eltrombopag and had at least 1 platelet count measurement in each of Cycles 1 and 2. Eltrombopag PK was analyzed and will be reported elsewhere.

## Results

### Patient demographics and disposition

**Figure 1:**
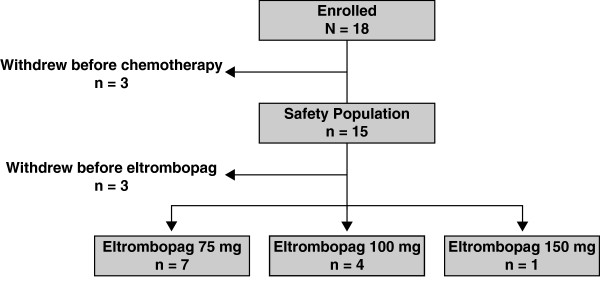
**Summary of Patient Disposition. **Of 18 patients enrolled, 3 withdrew before receiving any chemotherapy and 15 received at least 1 chemotherapy dose (safety population). Of these 15 patients, 3 withdrew before receiving a first dose of eltrombopag during Cycle 2. Seven, 4, and 1 patients received at least 1 dose of 75 mg, 100 mg, and 150 mg eltrombopag, respectively.

Due to slow patient recruitment over a 4-year period, enrollment into the study was ended prior to achieving sufficient patients to meet all predetermined study objectives, including identification of OBD and enrollment into an expansion phase. In addition, no evaluable doxorubicin PK samples were collected for assessment of the potential doxorubicin-eltrombopag PK interaction. A total of 18 patients were enrolled into the study. Three patients withdrew prior to receiving any chemotherapy and 15 patients received at least 1 dose of chemotherapy (safety population, Table 1). Of these 15 patients, 12 received at least 1 dose of eltrombopag: 7, 4, and 1 patients received 75 mg, 100 mg, and 150 mg eltrombopag daily, respectively (Figure 1). Two of the 7 patients who received 75 mg eltrombopag were treated for 10 days post AI chemotherapy; the remainder of patients received eltrombopag for 5 days prior to and 5 days post AI chemotherapy. Three patients within the safety population withdrew prior to eltrombopag dosing: 1 due to a serious AE (SAE, Grade 3 pulmonary embolism), 1 due to disease progression, and 1 due to patient decision. The median age (range) was 44 (20–65) years and 53% were male.

**Table 1:** Patient demographics and baseline clinical characteristics (safety population)

**Demographics**	**No**	**Eltrombopag**	**Eltrombopag**	**Eltrombopag**	**Total**
	**Eltrombopag**	**75 mg**	**100 mg**	**150 mg**	**(N = 15)**
	**(n = 3)**^**a**^	**(n = 7)**	**(n = 4)**	**(n = 1)**	
Median age, y (range)	56.0 (20–65)	48.0 (38–62)	30.5 (23–44)	59.0	44.0 (20–65)
Gender, n (%)					
Female	1 (33)	4 (57)	2 (50)	1 (100)	8 (53)
Male	2 (67)	3 (43)	2 (50)	0 (0)	7 (47)
Race, n (%)					
Hispanic or Latino	1 (33)	0 (0)	2 (50)	0 (0)	3 (20)
Not Hispanic or Latino	2 (67)	7 (100)	2 (50)	1 (100)	12 (80)
**Baseline clinical characteristics**					
Median baseline platelet count, 1000/μL (range)	256.0	300.0	264.0	388.0	281.0
	(218–371)	(197–368)	(180–595)		(180–595)
ECOG PS					
ECOG 0, n (%)	0 (0)	6 (86)	0 (0)	1 (100)	7 (47)
ECOG 1, n (%)	3 (100)	1 (14)	4 (100)	0 (0)	8 (53)

^a^Patients were withdrawn prior to receiving eltrombopag during the second cycle.

ECOG PS, Eastern Cooperative Oncology Group Performance Status.

During Cycle 2, 12 patients received a median (range) of 8.5 (2–17) days of treatment with eltrombopag; 7 (2–17), 8.5 (6–10), and 10.0 days for the 75-mg, 100-mg, and 150-mg dose groups, respectively. During Cycle 3, 9 patients received a median (range) of 10 (3–12) days of treatment with eltrombopag; 10.0 (3–12), 10.0 (7–10), and 10.0 days for the eltrombopag 75 mg, 100-mg, and 150-mg dose groups, respectively. During Cycle 4, 5 patients received a median (range) of 10 (3–10) days of treatment with eltrombopag; 10.0 (3–10) and 10.0 days for the eltrombopag 75 mg and 100 mg dose groups, respectively. During Cycle 5, 2 patients received a median (range) of 6 (2–10) days of treatment with eltrombopag; 2 and 10.0 days for the eltrombopag 75-mg and 100-mg dose groups, respectively.

### Safety

Since the study did not complete as planned, analyses of safety are limited. Five patients who received eltrombopag completed the study (ie, received at least 1 dose of eltrombopag and underwent all visits through to completion of Cycle 2). The majority of patients in the safety population (10/15, 67%) withdrew prior to study completion. Reasons for study withdrawal included AEs; loss to follow-up; disease progression; patient decision; poor tumor response to AI therapy; evaluation for surgical amputation; and inability to continue AI therapy.

All patients experienced at least 1 AE while enrolled in the study. Thrombocytopenia (12 patients, 80%), neutropenia (11 patients, 73%), and anemia (10 patients, 67%) were the most common hematologic AEs; and fatigue (8 patients, 53%), alanine aminotransferase (ALT) increased, constipation, and nausea (7 patients each, 47%) were the most common nonhematologic AEs (Table 2). Grade 3 and 4 toxicities occurring in each group are listed in Table 3.

**Table 2:** Adverse events of any grade (≥ 15% of patients, safety population)

**Treatment-emergent**	**No**	**Eltrombopag**	**Eltrombopag**	**Eltrombopag**	**Total**
	**Eltrombopag**	**75 mg**	**100 mg**	**150 mg**	**(N = 15)**
	**(n = 3)**^**a**^	**(n = 7)**	**(n = 4)**	**(n = 1)**	
Hematologic AEs, n (%)					
Thrombocytopenia	2 (67)	5 (71)	4 (100)	1 (100)	12 (80)
Neutropenia	2 (67)	5 (71)	4 (100)	0	11 (73)
Anemia	1 (33)	6 (86)	3 (75)	0	10 (67)
Leukopenia	0	3 (43)	2 (50)	0	5 (33)
Febrile neutropenia	0	2 (29)	1 (25)	1 (100)	4 (27)
Thrombocytosis	0	4 (57)	0	0	4 (27)
Nonhematologic AEs, n (%)					
Fatigue	0	6 (86)	2 (50)	0	8 (53)
ALT increased	0	5 (71)	2 (50)	0	7 (47)
Constipation	0	6 (86)	1 (25)	0	7 (47)
Nausea	1 (33)	5 (71)	1 (25)	0	7 (47)
Alopecia	0	5 (71)	1 (25)	0	6 (40)
Pyrexia	1 (33)	3 (43)	2 (50)	0	6 (40)
Vomiting	1 (33)	3 (43)	2 (50)	0	6 (40)
AST increased	0	3 (43)	2 (50)	0	5 (33)
Hypokalemia	0	3 (43)	2 (50)	0	5 (33)
Confusional state	1 (33)	2 (29)	0	0	3 (20)
Hemorrhoids	0	3 (43)	0	0	3 (20)
Hypocalcemia	0	1 (14)	2 (50)	0	3 (20)
Headache	0	3 (43)	0	0	3 (20)
Edema peripheral	1 (33)	1 (14)	1 (25)	0	3 (20)
Proteinuria	0	3 (43)	0	0	3 (20)
Vitamin B12 increased	0	3 (43)	0	0	3 (20)

^a^Patients were withdrawn prior to receiving eltrombopag during the second cycle.

AE, adverse events; ALT, alanine aminotransferase; AST, aspartate aminotransferase.

**Table 3:** Grade 3 or 4 adverse events (safety population)

**Treatment-emergent**	**No**	**Eltrombopag**	**Eltrombopag**	**Eltrombopag**
	**Eltrombopag**	**75 mg**	**100 mg**	**150 mg**
	**(n = 3)**^**a**^	**(n = 7)**	**(n = 4)**	**(n = 1)**
Hematologic AEs, n (%)				
Thrombocytopenia	0	3 (43)	2 (50)	1 (100)
Neutropenia	2 (67)	4 (57)	4 (100)	0
Anemia	0	3 (43)	2 (50)	0
Leukopenia	0	3 (43)	0	0
Febrile neutropenia	0	2 (29)	0	0
Nonhematologic AEs, n (%)				
Pulmonary embolism	1 (33)	0	0	0
Abdominal abscess	1 (33)	0	0	0
Abdominal pain	0	1 (14)	0	0
Mucosal inflammation	0	1 (14)	0	0
Dehydration	0	1 (14)	0	0
Subclavian vein thrombosis	0	1 (14)	0	0
Sepsis	0	0	1 (25)	0

^a^Patients were withdrawn prior to receiving eltrombopag during the second cycle.

AE, adverse events.

No AEs considered related to study treatment were reported for patients receiving 100 mg and 150 mg dosages of eltrombopag. Five patients in the 75-mg group had 20 AEs reported as related to eltrombopag dosing. Eltrombopag-related AEs occurring in ≥ 2 patients were thrombocytosis (3 patients), anemia, fatigue, and thrombocytopenia (2 patients each).

Overall, 11 SAEs were reported in 7 patients. The 2 patients who withdrew prior to receiving any eltrombopag experienced 3 SAEs. Four patients in the 75-mg group experienced 7 SAEs, 1 of which (subclavian venous thrombosis) was reported as related to eltrombopag dosing. One patient in the 100-mg group experienced 1 SAE (sepsis), which was reported as unrelated to eltrombopag. No SAEs were reported for the 1 patient who received 150 mg eltrombopag. No deaths were reported in this study.

Three patients reported 1 SAE each leading to permanent discontinuation or withdrawal: 2 patients in the 75-mg group and 1 patient who never received eltrombopag. One of these SAEs, the Grade 3 subclavian venous thrombosis described above, occurred in a patient in the 75-mg group with no prior history of TEEs; further details are included below.

Ten patients reached a platelet count > 400,000/μL on at least 1 occasion, requiring temporary interruption of eltrombopag per protocol; none of these platelet count increases were associated with sequelae. Platelet count increases occurred at various points throughout the cycle; no pattern was observed.

Three patients in the 75-mg group reported 6 bleeding AEs, all Grade 1. The 1 patient who received 150 mg eltrombopag experienced Grade 3 epistaxis (Cycle 3; proximal platelets 15,000/μL). No bleeding events led to discontinuation of eltrombopag dosing or study withdrawal, and none were considered by the investigator to be related to eltrombopag dosing. An additional patient who did not receive eltrombopag reported three Grade 2 bleeding AEs during Cycle 1 of AI chemotherapy: hematemesis (proximal platelets 371,000/μL), hematuria (proximal platelets 126,000/μL), and hemoptysis (proximal platelets 126,000/μL).

Two patients who received 75 mg eltrombopag and 1 patient who never received eltrombopag experienced TEEs during the study. One patient who did not receive eltrombopag experienced a Grade 3 pulmonary embolism 3 days after completion of the first cycle of chemotherapy; proximal platelet counts were 126,000/μL. The event resolved 18 days later. The 2 patients who received 75 mg eltrombopag both experienced a Grade 3 subclavian venous thrombosis at proximal platelet counts of 193,000/μL and 284,000/μL. The former event, as described above, was considered by the investigator to be possibly related to eltrombopag dosing. The investigator also considered that the event may have been due to a port insertion that was located on the same side as the event. The TEE resolved 6 months later. The latter patient had concurrent estrogen use and a prior history of a TEE (deep vein thrombosis [DVT]); the patient had been enrolled under a prior protocol amendment that excluded patients with prior TEEs. The patient was withdrawn from the study after 2 days of eltrombopag dosing and the TEE resolved 11 days later. This event was considered by the investigator to be unrelated to eltrombopag.

All hepatobiliary laboratory abnormalities (HBLAs) were Grade 1 or Grade 2, none were considered related to eltrombopag dosing, and none required permanent discontinuation of eltrombopag or study withdrawal.

No patient experienced renal events with onset during eltrombopag dosing or within 6 months post-treatment. All creatinine values were reported as normal at all assessments.

Four cardiac-related AEs (palpitations, 2 events; tachycardia, 2 events) were reported for 3 patients who received 75 mg of eltrombopag. All were Grade 1 in severity and all were considered unrelated to eltrombopag dosing. All but one event (tachycardia) resolved. No clinically significant ECG results were observed. All QTc values were ≤ 500 msec throughout the study. No clinically meaningful decrease in ejection fraction was reported for the 1 patient (in the 150-mg group) who completed MUGA/ECHO assessment.

No new cataracts or progression of existing cataracts was reported.

### Efficacy

**Figure 2:**
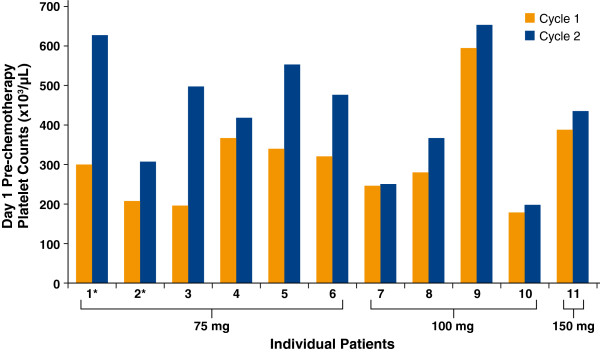
**Day 1 Pre-Chemotherapy Platelet Counts for Cycle 1 and Cycle 2 for Individual Patients Who Received Eltrombopag and Completed at Least 2 Cycles (n = 11).** Patients indicated with an asterisk (*) received eltrombopag for 10 days post-chemotherapy beginning in Cycle 2; all other patients received eltrombopag beginning in Cycle 2 for 5 days pre- and 5 days post-chemotherapy.

Since the study did not complete as planned due to poor enrollment, analyses of efficacy are also necessarily limited. Of the 12 patients who received at least 1 dose of eltrombopag 75 mg, 100 mg, or 150 mg, 11 patients had at least 1 platelet count measurement in each of Cycles 1 and 2 while on study and were evaluable for efficacy. Available data demonstrated increased pre-chemotherapy platelet counts on Day 1 of Cycle 2 (cycle with eltrombopag) compared to Day 1 of Cycle 1 (cycle without eltrombopag) in all 11 of these patients (Figure 2). Ten of these 11 patients received additional cycles of therapy (including eltrombopag) beyond Cycle 2; of these 10, 6 showed increased pre-chemotherapy platelet counts in all treatment cycles compared to Cycle 1 (5 in the 75-mg group and 1 in the 100-mg group). Two patients who were chemotherapy naïve (patients 1 and 2), and who did not have thrombocytopenia prior to study entry (ie, prior to the protocol change), had higher platelet counts on Day 1 of Cycle 2 than during Cycle 1. This is most likely due to natural rebound or recovery of hematopoiesis at the end of Cycle 1.

**Figure 3:**
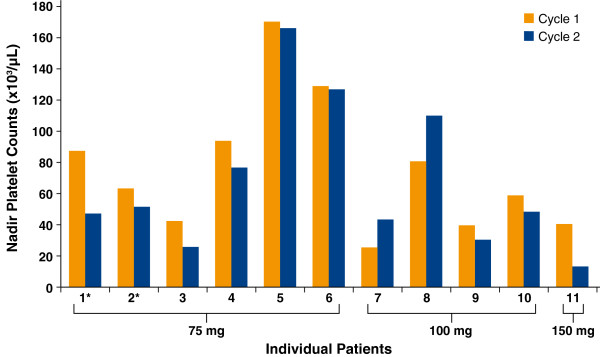
**Platelet Nadirs in Cycle 1 and Cycle 2 of Treatment for Individual Patients Who Received Eltrombopag and Completed at Least 2 Cycles (n = 11). **Patients indicated with an asterisk (*) received eltrombopag for 10 days post-chemotherapy beginning in Cycle 2; all other patients received eltrombopag beginning in Cycle 2 for 5 days pre- and 5 days post-chemotherapy.

Platelet nadirs for these 11 patients are shown in Figure 3. Two of 4 patients who received 100 mg eltrombopag daily demonstrated improved platelet nadirs in Cycle 2 (cycle with eltrombopag) compared to Cycle 1 (cycle without eltrombopag). The other 2 patients who received 100 mg eltrombopag daily did not receive their full 5 days of post-chemotherapy eltrombopag dosing.

## Discussion

Thrombocytopenia is a common side effect of chemotherapy, and multiple studies have suggested that CIT is a dose-limiting AE in the treatment of cancer, and can necessitate dose delays and/or dose reductions [23,24]. For example, a database analysis of 47,159 patients with both solid tumors and hematologic malignancies showed that TCP increased from 11% at baseline to 22-64% following chemotherapy treatment [24]. Grade 3/4 TCP has been reported in 63% of patients with advanced STS treated with AI chemotherapy [6]. An effective agent for the treatment of CIT may allow chemotherapy administration according to schedule and without dose delays and/or reductions.

This study evaluated the safety and efficacy of eltrombopag in patients with advanced STS and CIT due to receiving AI chemotherapy. Enrollment into this study was challenging as the target study population dwindled due to the emergence of novel standards of care for advanced STS during the 4-year course of the study. Despite implementation of several strategies to boost enrollment, patient recruitment remained slow and the study closed prior to recruitment of the planned number of patients.

Although data are limited, repeated treatment cycles of eltrombopag appeared to be generally well tolerated and the safety profile was consistent with the known safety profile of eltrombopag and with what is expected for patients with advanced STS receiving treatment with the AI chemotherapy regimen. TEEs were observed in this study in 1 patient who did not receive eltrombopag and 2 patients who received eltrombopag, consistent with the known propensity for TEEs in cancer patients. Both eltrombopag-treated patients had known risk factors for TEEs (a port insertion on the same side for one patient, and prior DVT with concurrent estrogen for the other). Renal and cardiac events were examined thoroughly and no eltrombopag-related renal or cardiac events of concern were reported. After this study was initiated, in vitro data showed eltrombopag was an inhibitor of the BCRP-mediated transport of cimetidine. Extensive cardiac safety assessments were subsequently implemented through a study amendment. More recent in vitro studies have shown the BCRP-mediated transport of doxorubicin is not inhibited by eltrombopag at concentrations up to 30 μM, the highest concentration that can be tested in vitro (unpublished data). This concentration is 3- to 4-fold higher than the Day 1 plasma eltrombopag concentration after administration of 100 mg eltrombopag on Days -5 to -1 (unpublished data). These observations suggest that the risk of a clinical eltrombopag-doxorubicin interaction may be far less than originally anticipated.

As shown in Table 2, 100% of patients treated at the 100 mg and 150 mg doses experienced TCP of any grade, whereas only in 71% of patients treated at the 75 mg experienced TCP. The main reason for this difference was that the protocol had no requirement for patients to have thrombocytopenia for study entry when patients were enrolling at the 75-mg dose level, and the patients enrolled at this eltrombopag dose were also chemotherapy naïve. The protocol was amended for patients enrolled at the 100-mg and 150-mg dose levels to require that the patient experience at least Grade 2 thrombocytopenia (platelets < 75,000/μL) prior to study entry. This resulted in patients who had previously received chemotherapy having a greater degree of thrombocytopenia at study entry, and explains the increased rate of TCP observed at the higher doses.

Although there was insufficient enrollment for identification of an OBD, no dose-limiting toxicities were observed that limited eltrombopag dose escalation to 150 mg daily in the study. The 75 mg eltrombopag dose demonstrated increased platelet counts at Day 1 of Cycle 2; however, this dose may be inadequate since it did not also improve platelet nadirs. Determination of the OBD for eltrombopag for patients with CIT requires further study.

Limited data were available to explore the efficacy of eltrombopag in this patient population. The study protocol required temporary interruption of eltrombopag dosing when a patient’s platelet counts were > 400,000/μL. Ten patients had platelet counts > 400,000/μL on at least one occasion, requiring temporary eltrombopag interruption; this may have decreased efficacy for these patients. Available platelet data showed that all patients receiving eltrombopag demonstrated increased pre-chemotherapy platelet counts during Cycle 2 (eltrombopag dosing cycle) compared to Cycle 1 (cycle prior to eltrombopag dosing); additionally, 6 of 10 patients who received > 2 cycles of therapy showed increased pre-chemotherapy platelet counts in each cycle compared to Cycle 1. Finally, 2 of 4 patients receiving 100 mg eltrombopag had improved platelet nadirs in Cycle 2 compared to Cycle 1. Both of these patients received eltrombopag 5 days pre- and 5 days post-chemotherapy, suggesting that this schedule might improve platelet nadirs as well as pre-chemotherapy platelet count, allowing patients to complete subsequent chemotherapy cycles at the planned dose and schedule.

Further studies are needed to better assess the effects of this pre- and post-chemotherapy schedule of eltrombopag administration in combination with other chemotherapy regimens. In an ongoing, randomized Phase I/II study of eltrombopag versus placebo in patients with solid tumors receiving gemcitabine alone or in combination with cisplatin or carboplatin (NCT01147809), eltrombopag 100 mg daily is being administered according to a pre- and post-chemotherapy schedule (5 days before and 5 days following Day 1 of chemotherapy). The results of this study will further clarify the safety and efficacy of this eltrombopag dosing schedule in combination with chemotherapy.

## Conclusions

Although data are limited, the safety profile was consistent with the known safety profile of eltrombopag and the AI chemotherapy regimen. These preliminary data suggest a potential pre- and post-chemotherapy dosing scheme for eltrombopag when administered with AI chemotherapy, and support further investigation of eltrombopag treatment in patients with CIT.

## Competing interests

Sant P Chawla receives honoraria and research funding/grants from, and provides consultancy for Merck, Ariad, Amgen, Threshold, Cytrax, and Berg. Arthur P Staddon and Andrew E Hendifar have no relevant financial relationships to disclose. Conrad Messam, Rita Patwardhan, and Yasser Mostafa Kamel are employees of and have equity ownership in GlaxoSmithKline (GSK).

## Authors’ contributions

SC and AH participated in the acquisition of and analyzed the clinical data. AS participated in the acquisition and interpretation of and analyzed the clinical data. He also contributed to the design of the amendments. CM contributed to the design of the study, and acquired, analyzed, and interpreted the data. RP is the statistician responsible for the design, analysis, and interpretation of the data. YMK is the medical monitor for the study; he has led amendments of the protocol, reviewed the data, and led the development of the clinical study report. All authors contributed to the writing and reviewing of the manuscript, and approved the final draft for publication.

## Pre-publication history

The pre-publication history for this paper can be accessed here:

http://www.biomedcentral.com/1471-2407/13/121/prepub
